# Supplementary material for: Developing Gut-Healthy Strains for Pets: Probiotic Potential and Genomic Insights of Canine-Derived Lactobacillus acidophilus GLA09
Source: Microorganisms. 2025 Feb 6;13(2):350. doi: 10.3390/microorganisms13020350 (PMC11858033; doi:10.3390/microorganisms13020350)
Supplement: Supplementary file 1 [file microorganisms-13-00350-s001.zip › microorganisms-3451798-supplementary.pdf]

Supplementary material

Table S1. Prediction of CRISPRs to *L. acidophilus* GLA09.

<table>
<tr><th>SeqID</th><th>Start</th><th>End</th><th>Score</th><th>Repetitive unit sequence</th></tr>
<tr><td>gnl|Bacteria|ctg_1</td><td>537913</td><td>539326</td><td>22</td><td>GTTTTTATTTAACTTTAGAGGAATGTAAAT</td></tr>
</table>

Table S2. Biogenic amine content of *L. acidophilus* GLA09.

Items	Content(ug/mL)
Putrescine	−
Tryptamine	−
Phenylethylamine	−
Cadaverine	−
Histamine	−
Tyramine	−
Spermidine	−
Spermine	−

BAs in the concentration range: <2 ug/mL (−), 2–10 ug/mL (+), 10–100 ug/mL (++) 100–1000 ug/mL (+++), >1000 ug/mL (++++).

Table S3. Inhibition test results of *L. acidophilus* GLA09.

Items	*E. coli*	*S. aureus*	*S.typhimurium*
GLA09	20.91±0.09	18.89±1.45	21.71±1.81
MRS (negative control)	-	-	-
CLP03(positive control)	18.74±0.80	17.10±1.04	16.09±0.42

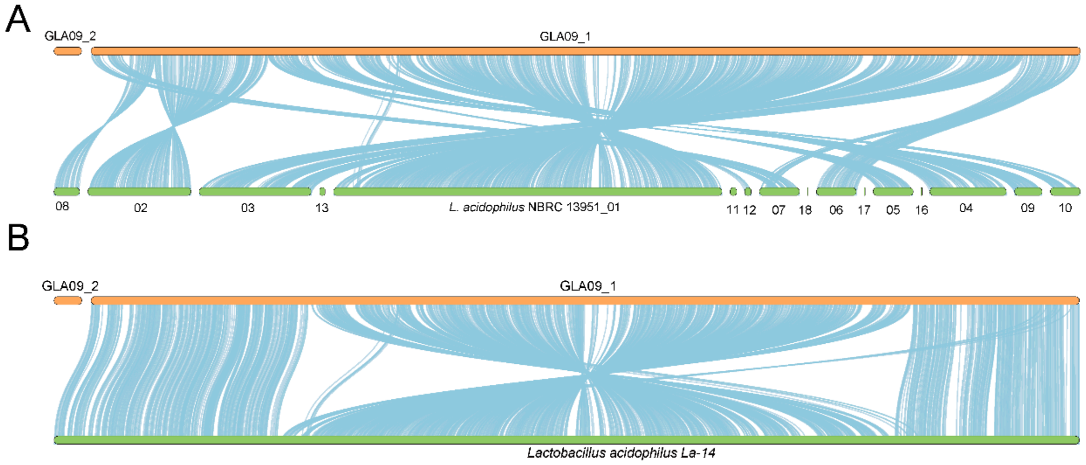

Figure S1. Comparative genomic covariance map of *L. acidophilus* GLA09.
